# Dilatation de bronches séquellaire d'une tuberculose pulmonaire au cours d'un syndrome de Rhupus 

**DOI:** 10.11604/pamj.2015.21.219.7421

**Published:** 2015-07-27

**Authors:** Olfa Berriche, Maher Dhifallah

**Affiliations:** 1 Service de Médecine Interne, Hopital Taher Sfar, Mahdia, Tunisie; 2 Service de Radiologie, Hôpital Mahdia Tunisie

**Keywords:** Lupus, polyarthrite, tuberculose, Lupus, polyarthritis, tuberculosis

## Image en medicine

La fréquence de la tuberculose est beaucoup plus élevée au cours des maladies systémiques que dans la population générale, il s'agit le plus souvent de la réactivation d'une tuberculose latente. A côté de l'immunodépression induite par la maladie sous-jacente, il faut souligner le rôle favorisant des glucocorticoïdes et des traitements immunosuppresseurs. Nous rapportons un cas de tuberculose pulmonaire compliquée d'une dilatation des broches (DDB) au cours d'une entité très rare appelée rhupus syndrome (lupus érythémateux systémique et polyarthrite rhumatoïde). Une patiente âgée de 41 ans suivie depuis 10 ans pour rhupussyndrome, elle était mise sous corticothérapie à fortes doses associée à des anti paludéens de synthèse et du méthotrexate. L’évolution ultérieure était marquée par l'amélioration de la symptomatologie cutanée et articulaire avec l'apparition d'une symptomatologie respiratoire trainante et récidivante. Le dernier épisode était marqué par une fièvre à 39°, une asthénie, un amaigrissement, une dyspnée et une toux. Le bilan tuberculeux était positif et la radiographie thoracique montrait un foyer de dilatation des bronches lobaire supérieur droit séquellaire d'une tuberculose pulmonaire. La patiente était mise antituberculeux avec bonne amélioration clinique et radiologique.

**Figure 1:**
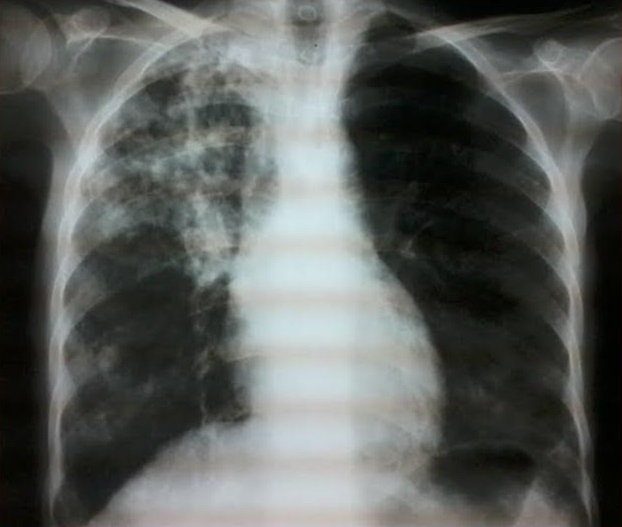
Foyer de dilatation des bronches séquellaire droit

